# A Sick Mind

**Published:** 1856-01

**Authors:** 


### A SICK MIND.

NOT a diseased mind; that might imply something akin to insanity; such is not our meaning. The body may be diseased functionally; the whole machinery is perfect as to parts, no portion is broken or destroyed, but it does not work well,—so we mean as to the mind, when we term it "sick;" it is perfect, it is sound, no decay, but it does not work smoothly, effectively. We all sometimes feel as to the body, a general tiredness, or discomfort, without being able to locate it precisely, or even give it a name, but we feel very sensibly that something is wrong; there is no elasticity, no vigor, no desire for activity. If under such circumstances we work at all, it is a dragging effort, it is an up-hill business, it is a labor; under different circumstances, work is an actual pleasure, and what we do tells; every stroke counts, and there is progress. Now apply this to the mind, and men who are accustomed to mental effort will feel our meaning at once. To accomplish much work physically, the whole powers of the body must be steadily engaged; but if called frequently away to do something else, or if only one hand be employed in the work proper, while the other is engaged in some secondary object, we see at once that a full day's work cannot be done. Precisely so with the mind. That man works efficiently, whose whole mental energies, undiverted, are laid out on the thing before him. Any one can make a practical experiment in this direction, by writing an hour alone in a private room, and writing another hour on a similar subject in his public office. There is not one of our thinking readers but sees our meaning, and feels its force. But, while all admit it theoretically, few indeed act upon it practically, as applied to an object, which is second in importance to none other known to man—we mean THE DIFFUSION OF PURE CHRISTIANITY. We beseech our readers to hear us patiently, for we argue *ad Hominum.*

This article has been suggested by an item in the *New York Independent* for November 1st, by a "MAINE SUBSCRIBER," on the *Retrogression* of "Congregationalism." In the next column is one of similar import from the *Christian Witness.* They intimate that the church is declining from want of the right kind of preaching. Other denominations have churches in various parts of the country, whose membership is not as large, nor influence as great, as it was twenty years or more ago in a population about the same. The churches with which we were ourselves familiar in boyhood, have nearly all dwindled as to influence, numbers and efficiency: and in New York city itself, several denominations number less, in proportion to the population, than they did many years ago, while the number of conversions a year, are most discouragingly few. We propose to show where the fault lies. Why is it that Bible Religion does not more rapidly widen in its influences? To advance any interest among men, there are three elements of success essential;

It must be important,
It must be practicable,
It must be strongly presented.

But as to Religion, nothing can be more "important," as all will allow.

It is "practicable" for all men to become religious, for *who-soever* WILL *let him come; ye* WILL NOT *come unto me that ye might have life*, is a "bone," or file, or fence rail, which the divine must gnaw, not the doctor; but the general idea is, that men could become religious, if they wanted to do so: hence, religion is not "impracticable," that it does not spread more rapidly must therefore arise from the manner of its presentation to the irreligious; there is where *we* trace the defect. Church members do not present it in their conduct, with that bold and beautiful distinctness which compels admiration and conviction. But this is not the main reason, this is not the point at which we are driving. As a doctor, we can find no rest, no starting point, no foundation,—until we discover THE *great cause!*

It is because the mind is sick, the mind of the ministers of religion is sick, so that they cannot present that great subject to men as it ought to be presented, and as never has failed of success in time past, nor ever can in time to come, when thus presented. The mind of the ministry is sick, because the people are at fault, in not affording the clergy that material aid, which, while it places them above all fear of want, at the same time releases them from all pecuniary care, and would enable them to concentrate, uninterruptedly, every energy of mind and heart in the great one work of urging upon men the first chief aim of life, due preparation for an immortal existence. What would become of any army, whose officers and men had to toil for their daily bread, or of any government whose leaders had to work for a living. What would be the results of any great national undertaking, if the energies of the leading men were constantly diverted or interrupted by necessary efforts to obtain and cook each daily meal. In every department of life, in every institution of society, involving responsibility, requiring talent, learning, cultivation, industry, men are paid for their management to an extent which enables them to spend their whole time, and their unfrittered energies in such management, and no undertaking can succeed, if its officials are not thus abundantly paid; there is not an instance of it in all history, and with these facts before us, it is past conjecture, except *from innate indifference*, how the common mind in this country has practically settled down upon the impression—that clergymen are to support themselves pecuniarily,—and being left to themselves thus, except an average pittance (out of cities and large towns) barely enough to feed a good horse, their capabilities are squandered in one way and another in efforts to provide food and clothing for their wives and children, while the surplus of time only is given to their appropriate calling; this indeed they are commanded to do, family first—the Church next, for if ANY man provide not for his household he is worse than an infidel. In the vain effort to give more of their time to the Church than their pecuniary situation allows, these excellent men, with all their learning, talents and capabilities, fall into embarrassments more wearing upon the bodily health, more wasting to the mental energies, than the hardest labor in the fields or on the highway. The medical reader of all schools will bear us out unhesitatingly, and without one dissenting voice, founded on the first principles of physical and mental science, that the human mind works efficiently in the proportion that it is exempted from all distracting influences. This subject merits the mature study of all Christian men, and then, the practical personal application as involved in the question,—*Is* MY *Minister's salary fully adequate to his whole family expenditure?*

